# Complex presentations of child conduct problems: Validation of a competency‐based model for clinical practice

**DOI:** 10.1002/jcv2.70045

**Published:** 2025-09-01

**Authors:** Jessica M. Barker, David J. Hawes

**Affiliations:** ^1^ School of Psychology University of Sydney Camperdown New South Wales Australia

**Keywords:** case complexity, clinical competence, disruptive, impulse control, and conduct disorders, evidence‐based practice, psychosocial intervention, therapeutic alliance

## Abstract

**Background:**

Children with conduct problems often present with a range of complex needs and many factors have the potential to complicate the delivery of evidence‐based interventions for conduct problems. Little, however, is known about how to optimise the delivery of such interventions for complex cases, and there has been a lack of consensus regarding the features that reflect case complexity in treating child conduct problems. The aims of this study were to examine practitioner perceptions of the factors that contribute to case complexity in child conduct problems, and perceptions of the therapist competencies necessitated by distinct features of case complexity when delivering these interventions.

**Methods:**

Practitioners (*n* = 49) with expertise in evidence‐based parenting interventions for conduct problems were recruited from nine countries. Using the Delphi method, participant consensus was established across multiple rounds of consultation and feedback with the expert panel.

**Results:**

Experts reached consensus on a model comprising seven distinct drivers of case complexity among children with conduct problems, spanning child‐related factors, caregiver‐related factors, and broader ecological factors. Consensus was also reached regarding 16 therapist competencies of key importance to distinct drivers of case complexity, thereby validating the conceptualisation of these drivers.

**Conclusions:**

These findings represent a novel consensus‐based practitioner perspective on the complex needs of children with conduct problems, and offer researchers and practitioners a common language for communicating information about case complexity. This perspective has the potential to inform clinical research, practice, and training, to better meet these complex client needs.

## Key Points

**What's known?**

- Parenting interventions based on social learning theory are the treatment of choice for child conduct problems, yet many factors may complicate the delivery of these interventions.
- Children with conduct problems often present with a range of complex needs, yet there has been a lack of consensus regarding the features that reflect case complexity among such children.

**What's new?**

- Expert practitioners across nine countries were able to reach consensus regarding the factors that contribute to case complexity among children with conduct problems, and some therapist competencies necessitated by distinct drivers of case complexity.
- Support was found for seven drivers of case complexity among children with conduct problems, spanning child‐level, caregiver‐level, and ecological factors.

**What's relevant?**

- Evidence regarding the practitioner perspective on complex presentations of child conduct problems is particularly novel, and has the potential to inform clinical practice and training guidelines for the delivery of evidence‐based interventions in real world settings.

## INTRODUCTION

Evidence‐based interventions for child conduct problems (e.g., oppositional and aggressive behaviour) are widely available, yet many factors may complicate the delivery of these interventions, and treatment for complex presentations of conduct problems remains poorly understood (Andrade et al., 2022; Fonagy & Luyten, 2018). At the same time, approaches to classifying such presentations as complex have often been inconsistent, thereby hampering progress. In the broader biomedical literature, *case complexity* has generally been defined as the presence of factors that make the delivery of treatment difficult (e.g., Kazdin & Whitley, 2006; Ruscio & Holohan, 2006; Safford et al., 2007; Schaink et al., 2012). It has been theorised that this complexity arises from a cumulative interplay between factors across multiple domains, spanning not only diagnostic features, but also environmental, socioeconomic, and cultural factors (Delgadillo et al., 2017; Shippee et al., 2012). Such a conceptualisation suggests that although the factors that drive case complexity may increase risk for poor treatment outcomes, nonresponse is not inevitable when case complexity is present. Importantly, optimal outcomes may depend in part on practitioner adaptations based on such factors (Georgiadis et al., 2020).

Multiple approaches to characterising presentations of conduct problems can be found in the literature. Diagnostic models of oppositional defiant disorder (ODD) and conduct disorder (CD) have evolved in recent years to account for this heterogeneity and emphasise multiple factors associated with increased risk. This includes the introduction of specifiers (e.g., irritability‐anger in ICD‐11), subtypes (e.g., early‐onset CD in DSM‐V‐TR), and severity classifications (e.g., for ODD based on number of problem contexts in DSM‐V‐TR). These models assume that clinical needs of children vary depending on such distinct presentations, based in part on evidence of treatment outcomes associated with risk pathways (Fairchild et al., 2019; Hawes et al., 2023). For example, the conduct problems of children with limited prosocial emotions are particularly severe and likely to persist following intervention, and novel treatment components for this group have therefore been subject to growing research (Perlstein et al., 2023).

Clinical presentations of conduct problems have also been described as complex due to factors beyond those specified within diagnostic models of ODD and CD. Most frequently, case complexity often refers to comorbid psychopathology (e.g., Dadds et al., 2012; Leijten et al., 2020; Weisz et al., 2012). Beyond comorbidity, clinical needs of children with conduct problems may also be complicated by contextual factors, which may impact adversely on treatment implementation and engagement, placing additional demands on practitioners. This includes caregiver‐related factors, such as quality of parenting, parental psychopathology, and factors in the broader ecology of the child, such as cultural fit of the programme, socioeconomic disadvantage, and other environmental stressors (Andrade et al., 2022; Chacko et al., 2016; Piotrowska et al., 2017).

Despite the broad range of factors implicated in complex needs of families presenting with child conduct problems, research has often operationalised such complexity on the basis of a single factor alone and has typically focussed on individual factors in relative isolation. Based on treatment outcome research and clinical experience, Hawes and Dadds (2021) argued that the needs of children with complex presentations of CPs are best represented across multiple domains. They proposed that the factors that contribute to case complexity among children with CPs relate largely to six dimensions. These dimensions reflect complexity arising from (1) the topography of the child's conduct problems (e.g., range and severity; settings occurring); (2) developmental and dispositional factors (e.g., language impairments; temperamental factors including callous and unemotional traits); (3) co‐morbid child psychopathology (e.g., internalising disorders; neurodevelopmental disorders); (4) quality of parenting (e.g., harsh or inconsistent discipline; parenting skill deficits); (5) parental characteristics (e.g., personality; mental health; parental attributions); and (6) the family system and social environment (e.g., interparental conflict; lack of social support; financial disadvantage). However, this model is yet to be tested empirically, and research continues to operationalise complex needs heterogeneously.

Variable definitions of *case complexity* in the literature hamper understanding of it and concerns have arisen amongst practitioners that complex client needs are not met by existing evidence‐based parenting interventions for child conduct problems. A major criticism of treatment outcome research in child and adolescent mental health is that it has often been conducted under controlled conditions that may not reflect real‐world clinical populations and settings, and this has resulted in a gap between research and practice related to factors of this kind (Ruscio & Holohan, 2006; Weisz et al., 2015). Indeed, lack of programme fit with client and service needs and practitioner beliefs has been found to be a core barrier to use of evidence‐based parenting interventions (Turner et al., 2011). Many therapists consider that overly rigid adherence to a manualised intervention detracts from therapeutic alliance or ability to respond to complexity arising during session (Addis & Krasnow, 2000; Chorpita et al., 2014). Literature has discussed that practitioners subsequently often perceive that evidence‐based interventions would not benefit their clients with complex needs, and that they may be more likely to rely on clinical judgement to determine how to meet these needs, which may be detrimental to clients (Ruscio & Holohan, 2006). Adaptation to client needs is recognised as necessary and common in community settings (Pinto et al., 2024), yet in the absence of guidelines concerning if and how practitioners should adapt programmes, and what competencies therapists require to do so, many adaptations deviate from the evidence base (Moore et al., 2013), broadening the research‐practice gap.

In the field of conduct problems, emerging research has targeted this gap by informing approaches to adapting evidence‐based interventions based on the unique needs of a case (for a review see Andrade et al., 2022). Research aimed at decreasing the research‐practice gap has included: examination of treatment effects associated with specific intervention elements or components (Garland et al., 2008; Leijten et al., 2019); development of transdiagnostic treatment components and modular models of delivery to address individual client needs related to complex comorbidities (Chorpita et al., 2005; Evans et al., 2021; Weisz et al., 2012); development of a framework for adaptations (Georgiadis et al., 2020); and addressing caregiver factors affecting clinical engagement and benefit (Gonzalez et al., 2023; Jones et al., 2021). Such approaches have shown much promise in translational research. For example, the Modular Approach to Therapy for Children (MATCH; Chorpita & Weisz, 2005) has been found to improve both client outcomes and efficiency of services compared to treatment as usual (Chorpita et al., 2017; Merry et al., 2020; Weisz et al., 2012). Research into MATCH has highlighted the importance of better understanding therapist competencies to support optimal intervention delivery (Cecilione et al., 2021).

One novel and potentially important approach identified to further reduce this research‐practice gap is to investigate practitioner perceptions of case complexity. Little is currently known about practitioner perceptions of the factors that contribute to case complexity among children with CPs, in terms of the specific features of a case that are likely to complicate the delivery of evidence‐based intervention and potentially cause practitioner deviation from evidence‐based practice. These perceptions may be particularly important to understanding the demands that case complexity places on practitioners, and to understanding therapist competencies for the optimal delivery of evidence‐based interventions with case complexity.

In implementation science, therapist competence has been conceptualised as a subcomponent of fidelity. Specifically, fidelity has been defined as comprising both adherence (i.e., implementation of programme components as intended according to protocol) and competence (i.e., the quality with which this is done, including therapist skill and style of delivery) (Breitenstein et al., 2010; Martin et al., 2023). Notably, a recent systematic review found that fidelity was most consistently associated with parenting intervention outcomes when operationalised in terms of adherence, competence and knowledge, compared to studies in which it was operationalised only in terms of adherence or another single component (Basha et al., 2025). Associated outcomes included improved parenting behaviours and stress, and improved child behaviour, self‐esteem, and adjustment. In addition to highlighting the significance of fidelity, this supports the importance of ongoing research into competence regarding the delivery of such interventions.

Therapist competencies for evidence‐based practice have been subject to growing research in their own right across various fields of mental health. Drawing on the expert consensus Delphi method used to inform the Improving Access to Psychological Therapies (IAPT) initiative in the UK (now called NHS Talking Therapies; Clark, 2018), Barker and Hawes (2024) produced a comprehensive model of competencies for the evidence‐based treatment of conduct problems. Such treatment was found to rely on a broad range of competencies, including 25 competencies related to general clinical practice in child mental health (e.g., knowledge of influence of cultural and racial factors on therapy); 27 specific to parenting interventions (e.g., use of experiential strategies to teach skills); and 23 specific to the content of evidence‐based interventions for conduct problems (e.g., nonviolent disciplining; caregiver‐child relationship enhancement; Barker & Hawes, 2024).

Therapist competencies may be particularly important to address complexity in treatment delivery. Practitioner perspectives on specific dimensions of case complexity, and what competencies may be particularly important to each, have not previously been examined. If practitioners perceive that case complexity has distinct dimensions, investigation of the specific competencies that are particularly important to each is also relevant, as these may differ between each and have implications for treatment adaptations. Whilst the term *case complexity* is used here for consistency with existing literature (Delgadillo et al., 2017; Hawes & Dadds, 2021; Kazdin & Whitley, 2006), we wish to clarify that our use of the term refers to difficulties clinicians face due to the limitations of current clinical practices, rather than difficulties that are inherent in a client. The major aims of the current study were therefore twofold. Firstly, we aimed to examine practitioner perceptions of the factors that contribute to case complexity among children with conduct problems. Specifically, we tested whether the model of key dimensions of case complexity proposed by Hawes and Dadds (2021) was supported by consensus among practitioners with high levels of experience in the delivery of evidence‐based parenting interventions for CPs. Secondly, we examined practitioner perceptions of the therapist competencies necessitated by distinct features of case complexity when delivering these interventions. Of key interest was the relative importance that experienced practitioners place on specific competencies (Barker & Hawes, 2024).

## METHODS

### Participants

Participants consisted of an international expert practitioner panel, all currently practicing a social learning‐based parenting intervention for conduct problems (hereby referred to as PIs), with at least 5 years of experience in PIs. 117 practitioners were invited by email, identified through relevant literature publications and recommendations from other practitioners who were recruited, including developers of established programmes. All interested eligible practitioners willing to complete the multiple rounds of data collection required (60 practitioners) were recruited with informed consent, with 49 participants completing the first round of data collection. The final sample, which was the same sample as that recruited for the Delphi study reported by Barker and Hawes (2024), was demographically highly comparable to those initially approached regarding sex, nationality, profession, and research experience. Forty one participants completed all three main testing rounds, resulting in a high participant retention rate (84%) compared to similar Delphi studies (Morrison & Barratt, 2010; Spain & Happé, 2020). Twenty‐eight participants completed the follow‐up round (57% retention rate)—a comparable retention rate to similar Delphi studies that had fewer testing rounds (Bauer et al., 2019; Kim et al., 2021; Rubio et al., 2020), highlighting strong participant engagement.

Participants represented nine countries across three continents, including the United States of America (51% of participants), Australia (31%), Canada, New Zealand, England, Wales, Germany, Finland, and the Netherlands. Professions were predominantly psychology (73% of participants) and social work (10%), along with nursing, education, and psychiatry. Clinical experience spanned the major PIs for conduct problems (e.g., Parent‐Child Interaction Therapy, 45% of participants practicing; Helping the Noncompliant Child, 24%; Triple‐P, 24%; Incredible Years, 22%; Parent Management Training‐Oregon Model, 22%; Barkley's Defiant Child, 16%; Parent‐Child Care, 14%). Practitioners had a mean of 18 years of experience in PIs, and 98% of participants reported working with case complexity in their current and/or previous positions. Varied settings were represented, including private practice, university/research clinics, community health settings including hospital‐based care, and government child welfare agencies. All participants provided informed consent to participate and chose whether to be identified (see acknowledgements for names of identified participants).

### Design

Practitioner perceptions regarding complex presentations of child conduct problems were examined through Delphi method consultation with the international expert panel. Data collection comprised three main testing rounds, undertaken in April‐May, June‐July, and September‐October of 2022, with a follow‐up round in July 2023 specifically to further examine competencies for complexity. Each round consisted of an approximately 30–50‐min questionnaire, for which participants were given two weeks to respond. Following Delphi method guidelines, consensus on proposed drivers of complexity, and competencies most vital for each, was developed through iterative questionnaire rounds in which participants provided qualitative responses and quantitative ratings (Chalmers & Armour, 2019). Participants remained anonymous to each other, whilst receiving summary feedback on panel responses in previous rounds, to encourage convergence of opinions. Participants could participate via videoconference (Zoom) interviews or via online questionnaires using the Qualtrics platform, whereby most chose the latter. Ethics approval was obtained from the University of Sydney Human Ethics Research Committee.

### Procedure

The first round of testing consisted of three parts: (1) participant revisions to a preliminary model of core therapist competencies for PIs for conduct problems (reported elsewhere; Barker & Hawes, 2024); (2) participant revisions, including edits, additions, and omissions, to a proposed model of drivers of case complexity for conduct problems (Hawes & Dadds, 2021); and (3) participant perceptions of what therapeutic competencies (from the proposed core competencies model or otherwise) they would draw on most, or see as most vital, to address each case‐complicating dimension. These priority competencies for case complexity were developed alongside the model of drivers of case complexity to minimise testing rounds and hence optimise participant retention. In doing so, participants were asked to consider the term *complex* throughout the survey to mean ‘particularly challenging, demanding, or difficult to treat’.

Following Round 1, revisions were made to the model of case complexity for conduct problems by content analysis of participant responses. Round 2 focussed on obtaining participant perspectives on these revisions. Participants were asked to select whether they perceived further revisions necessary for each part of each model. Consensus support was received for the model of drivers of case complexity in Round 2 hence no further revisions were required. Round 3 therefore focussed on developing consensus on priority competencies specific to each driver of case complexity. For each driver, participants were presented with the priority competencies suggested in Round 1 and asked to identify those they perceived as most vital. A follow‐up, Round 4, was implemented to refine consensus for priority competencies as consensus was not met in Round 3. In Round 4, participants were again asked to select which competencies they perceived as most important for each driver of case complexity and additionally, of those, rank their order of importance in addressing that driver. For each driver of case complexity, participants were informed of which competencies met consensus in the previous round, and which did not have agreement.

Content analysis of open‐ended expert responses to Round 1 was conducted manually, to aptly revise the drivers of case complexity. Reliability of analysis was evidenced by the researchers' agreement on content themes, drivers of case complexity, and wording of final competencies. Participant acceptability of the revised model showed the analysis had good validity (Chalmers & Armour, 2019). Data was deidentified to minimise bias. The criterion for consensus, consistent with recent Delphi studies, was 70% agreement for each individual driver of case complexity (Khazaie & Khan, 2020; Kim et al., 2021). For each priority competency, consensus required at least 70% of participants to both (a) rate the competency as ‘most’ vital; and (b) rank the competency amongst the number most highly rated. This double consensus method was employed to optimise validity of consensus.

## RESULTS

### Drivers of case complexity

Practitioners reached consensus regarding the factors that contribute to complex presentations of child conduct problems, and the grouping of these factors into seven dimensions, or drivers, of case complexity (see Table 1). Ninety four percent of experts agreed that the six‐driver model proposed by Hawes and Dadds (2021) was acceptable, that is, that it appropriately grouped factors associated with case complexity in child conduct problems. However, in Round 1, almost half of experts (42%) suggested revisions to this model. Consequently, each driver, except severity of child conduct problems, was revised by content analysis.

**TABLE 1:** Drivers of and priority therapist competencies for case complexity in conduct problems.

Driver of complexity	Priority therapist competencies
Severity of child conduct problems The frequency and range of problem behaviours/symptoms, and the range of settings in which symptoms create impairment (e.g., home, school, with peers)	**No specific competencies were identified as particularly important above other competencies.**
Child developmental history Developmental history as typically gathered in clinical assessment or reported in medical documentation. This includes language impairments, traumatic experiences etc	**For treatment planning:** Devise, implement, and revise treatment plans, collaboratively and flexibly, especially adjusting for child factors such as age, developmental needs, ability level and other factors (e.g., through additional modules/content)
Comorbid child psychopathology and dispositional factors Temperament, irritability, callous‐unemotional traits, emotional dysregulation, internalising disorders like anxiety, depression and PTSD, neurodevelopmental disorders like ADHD	**For assessment:** Assess symptoms, developmental factors, and function of behaviour (functional analysis) including those related to comorbidities (e.g., self‐injurious or repetitive behaviours in ASD), trauma history, and family history, to identify most appropriate intervention(s) based on the child's needs
	**For treatment planning and delivery:** Collaboratively and flexibly devise, implement, and revise treatment plans, helping the family prioritise the most significant concerns to address first in therapy, applying all parenting intervention skills in accordance with the needs of the individual child, to address what is possible for each presenting issue
	**For assessment, case formulation and goal setting:** Use skills for evidence‐based multi‐method, multi‐informant assessment to be very clear on the case formulation, diagnoses (including primary/secondary and disorder subtypes) and what factors are maintaining the problems, to therefore be clear on primary diagnoses or needs and consequent treatment goals and priorities
Caregiver‐child relationship and parenting practices Significant lack of: warmth, engagement, positive affect, and sensitivity in caregiver‐child relationship; permissive, harsh, or inconsistent discipline; parenting skill deficits	**For therapeutic relationship:** Build a collaborative therapeutic relationship with primary caregivers and repair any ruptures (e.g., by focusing on building rapport, showing empathy, seeking to understand the primary caregivers and prevent potential negative countertransference, and potentially engage them with additional techniques such as motivational interviewing); so that primary caregivers see the therapist as an ally, are willing to collaborate and build investment in the intervention including shared goals, and also can be challenged in their parenting without becoming defencive
	**For barriers to change:** Support primary caregivers to overcome barriers to treatment and manage resistance to change (e.g., assess, formulate and manage barriers to engagement, including relational dynamics; apply motivational interviewing/motivational enhancement strategies)
	**For assessment:** Assess quality and patterns of parenting and caregiver‐child interaction, and drivers thereof, using valid methods of observation, interview and ongoing sessions
	**For strengths‐based practice:** Apply culturally responsive and strengths‐based practices (e.g., acknowledging the unique experiences of families; recognising primary caregivers as experts on their child)—including to reduce negative countertransference
	**For goal setting:** Collaboratively create and set concrete and measurable shared treatment and session goals with caregivers to encourage caregiver investment in the intervention and alignment with the therapist, even when addressing parenting deficits
Parental characteristics Parental personality, mental health (e.g., depression, substance use), trauma history, or cognitive/affective processes (e.g., attributional biases, emotional flooding, poor self‐regulation)	**For therapeutic relationship:** Develop and maintain a trusting therapeutic relationship with primary caregivers, with rapport and empathy, developing this before moving into active intervention, to engage primary caregivers as allies, gain access to information about family dynamics, reduce negative countertransference, and effectively intervene in parenting
Family context Family structure and dysfunction (e.g., interparental conflict, parenting conflict with extended family); parental social and economic adversity (e.g., lack of social support, financial disadvantage); cultural beliefs, context and connectedness; intergenerational trauma; treatment history and experiences	**For therapeutic relationship:** Foster and maintain engagement with primary caregivers in a collaborative therapeutic alliance (e.g., identifying and inviting in the child's parenting team; managing in‐session conflict between caregivers; managing resistance and ruptures, including client challenges to the competence of the therapist)
	**Culturally responsive and strengths‐based practice:** Apply culturally responsive and strengths‐based practices, including recognising, understanding and clarifying the family's unique perspectives, experiences and knowledge; integrating the value, role and influence of culture for the family into formulation, psychoeducation and discussions; and adapting interventions and specific techniques with cultural sensitivity and humility so that they're in line with the family's cultural beliefs and values
	**For assessment:** Conduct a thorough and broad assessment (including of intergenerational or historical trauma; family storyline; social support; the family's ability to meet basic needs; culture; parental beliefs and cognitions; family dynamics such as responses to child behaviour; and other psychosocial factors), to understand the role of the broader context (including strengths and barriers) in the child's presenting problems and develop realistic treatment expectations for planning and outcomes
Broader social environment Historical and/or systemic oppression, systems involvement for the family (e.g., child protective services, juvenile justice, parental justice involvement)	**For assessment:** Conduct a thorough and broad assessment (including of intergenerational or historical trauma; family storyline; social support; the family's ability to meet basic needs; culture; parental beliefs and cognitions; family dynamics such as responses to child behaviour; and other psychosocial factors), to understand the role of the broader context (including strengths and barriers) in the child's presenting problems and develop realistic treatment expectations for planning and outcomes
	**Culturally responsive and strengths‐based practice:** Apply culturally responsive and strengths‐based practices, including recognising, understanding and clarifying the family's unique perspectives, experiences and knowledge; integrating the value, role and influence of culture for the family into formulation, psychoeducation and discussions; and adapting interventions and specific techniques with cultural sensitivity and humility so that they're in line with the family's cultural beliefs and values
	**For therapeutic relationship:** Foster and maintain engagement with primary caregivers in a collaborative therapeutic alliance (e.g., identifying and inviting in the child's parenting team; managing in‐session conflict between caregivers; managing resistance and ruptures, including client challenges to the competence of the therapist)

*Note*: Competencies are ordered by those most frequently ranked most highly by the panel listed first for each driver.

Abbreviations: ADHD, attention‐deficit/hyperactivity disorder; PTSD, post‐traumatic stress disorder.

The final model of drivers contributing to complicating the delivery of PIs for child conduct problems consisted of seven drivers. Three drivers of case complexity concerned child‐level factors: (1) conduct problem severity; (2) developmental history; and (3) comorbid child psychopathology and dispositional factors. Additionally, two drivers related to caregiver‐level factors: (4) the caregiver‐child relationship and parenting practices, and (5) parental characteristics; and two drivers related to ecological factors: (6) family context (e.g. interparental conflict; cultural beliefs such as those that stigmatise mental health); and (7) broader social environment (e.g., legal system involvement; other systems involvement).

Experts reached consensus that factors related to parental characteristics, family context, and the broader social environment, always or almost always contributed to complexity among cases of conduct problems in early‐to‐middle childhood. Notably, practitioners also reached consensus regarding the cumulative nature of these drivers as they relate to overall level of complexity, with 94% of participants agreeing or strongly agreeing that ‘when more of these factors/dimensions are present, a case is likely to be more complex’. The remaining 6% of participants responded ‘neutral’; none disagreed.

### Priority competencies for case complexity

Expert practitioners also reached consensus on up to five competencies for each driver of case complexity that they endorsed as particularly important to achieve optimal outcomes when that driver is present (see Table 1). 508 priority competencies were initially identified by participants across the respective drivers of case complexity in Round 1. Following Round 4, experts reached consensus on 16 priority competencies across the drivers. Notably, consensus was not reached on all competencies, with additional competencies identified as important by 30%–70% of participants. However, further rounds to reach more consensus were not possible due to attrition (Hasson et al., 2000; Sumsion, 1998).

The priority competencies identified by practitioner consensus for each driver of case complexity (Table 1) were adapted from those in the model of core therapist competencies by Barker and Hawes (2024). Of note, all priority competencies concerned generic therapeutic competencies and process skills/knowledge rather than specific parenting skills and techniques, and no priority competencies were identified for complexity driven by conduct problem severity. Competency regarding treatment planning was identified as important for the remaining two child‐level drivers: for developmental history, this competency included adjusting treatment plans for child factors including age, developmental needs, intellectual ability and other factors; whereas for comorbid child psychopathology and dispositional factors, this included helping the family prioritise the most significant concerns. Additional priority competencies were also identified for complexity driven by comorbidity and dispositional factors but not for developmental history. For each driver of case complexity beyond these child level‐factors, a priority competency regarding the therapeutic relationship was identified. Specifically, a focus on building the therapeutic relationship with primary caregivers before moving into active intervention was the only priority competency highlighted by practitioners for complexity driven by parental characteristics. For other drivers, additional priority competencies were also identified alongside those specific to the therapeutic relationship. Priority competencies for ecological drivers of case complexity—family context and broader social environment—concerned assessment; culturally responsive and strengths‐based practice; and the therapeutic relationship.

## DISCUSSION

Parenting interventions (PIs) for conduct problems are amongst the most well researched interventions in child and youth mental health, yet they appear to often fall short of meeting the complex needs of children and families. Little is known about practitioner perceptions of the factors that contribute to case complexity among children with conduct problems (i.e., factors that make the treatment delivery process more difficult), or the therapist competencies necessitated by distinct features of case complexity. The current study is the first, to our knowledge, to examine practitioner consensus regarding these factors.

A model of drivers of case complexity originally proposed by Hawes and Dadds (2021), based on treatment outcome research, was acceptable to practitioners yet was found by the majority of participants to need revision, showing that it is not the model of best fit according to practitioners. The novel model produced here differs in both number and composition of these drivers and reflects consensus among expert practitioners from diverse practice settings and interventions, across nine countries. The current model therefore integrates treatment outcome evidence with the practitioner perspective, to provide a novel account of case complexity among children with conduct problems. These drivers of complex client needs include factors that complicate the treatment process but may not have a negative effect on outcomes of PIs, and, in the case of severity, may indeed have a positive effect on child conduct problem outcomes (e.g., Leijten et al., 2020), indicating that case complexity does not equate to nonresponse if intervention is delivered competently. Our findings also build on previous research regarding practitioner competencies for the delivery of evidence‐based interventions for conduct problems (Barker & Hawes, 2024), by identifying specific therapist competencies of key importance for each distinct driver of case complexity.

The first three drivers in this novel model of case complexity in child conduct problems relate to child‐level factors: (1) conduct problem severity; (2) developmental history; and (3) comorbid child psychopathology and dispositional factors. These largely reflect child‐level features specified in current approaches to classifying disruptive behaviour disorders in DSM‐5 and ICD‐11, including diagnostic specifiers for high‐risk subtypes therein. Interestingly, practitioners endorsed a model in which child characteristics such as irritability and callous‐unemotional traits were represented on a dimension incorporating comorbid child psychopathology and dispositional factors, separate to a developmental history dimension. This represents a modification to the original Hawes and Dadds (2021) model, and is consistent with qualitative responses from participants indicating a perception that the implications of such characteristics for treatment are often more considerable than those associated with common risk factors in a child's developmental history (e.g., language delays), and that they can be difficult to distinguish from comorbid symptoms in clinical practice. This is also consistent with transdiagnostic accounts of these characteristics (Chin et al., 2025; Hawes et al., 2023).

Two caregiver‐level drivers in the model of case complexity comprised (4) caregiver‐child relationship and parenting practices; and (5) parental characteristics. This is consistent with literature showing that caregivers play a critical role as agents of change in evidence‐based interventions for conduct problems, and that both the caregiver‐child relationship and specific parenting techniques contribute to change processes in these interventions (Leijten et al., 2018). The final two drivers in the current model, family context and broader social environment, further highlight a distinction between case complexity arising from these two factors, which were considered one dimension in the previously proposed model (Hawes & Dadds, 2021). These have both previously been found to impact adversely on engagement with clinical services and the translation of treatment delivery into enduring change in PIs (Piotrowska et al., 2017; Weisenmuller & Hilton, 2021).

In addition to characterising distinct types of factors that serve as drivers of case complexity, our results support a cumulative perspective on the contribution of these drivers to case complexity, consistent with models of complexity in physical health literature (e.g., Shippee et al., 2012). This does not preclude the possibility that a case may present as highly complex based on factors within a single domain (e.g., multiple comorbidities). Importantly, however, the emphasis here on high case complexity often spanning distinct risk‐factor domains diverges considerably from research in which such complexity has been operationalised based on a single factor alone (e.g., comorbidity; Aitken et al., 2018; Dadds et al., 2012), or research that investigates multiple drivers of case complexity but neglects to explore the cumulative effect of these (e.g., Kazdin & Whitley, 2006; McMahon et al., 2021).

It is noteworthy that practitioners working in diverse settings demonstrate consensus regarding some of the competencies of distinct importance for responding to specific drivers of case complexity. Our findings can be seen to validate the distinction between the respective drivers in the current model, and to support the utility of this distinction in clinical practice. However, practitioner consensus on priority competencies for case complexity was not reached on all competencies in the current study, suggesting diversity among practitioners' responses to case complexity. This supports the need for further research, including implementation research (Pinto et al., 2024), and more specific frameworks for how and when to use adaptations (Georgiadis et al., 2020).

It is noteworthy that no priority competencies were identified for complexity arising from severity of child conduct problems. This suggests that practitioners may perceive all core competencies as equally important for highly severe presentations, consistent with literature showing that children with severe conduct problems benefit particularly well from existing PIs as delivered in standard practice (Leijten et al., 2018), and that adapting these interventions beyond their core components of caregiver‐child relationship enhancement and effective non‐violent discipline may lead to reduced benefit for severe conduct problems (Leijten et al., 2022). Regarding other child‐level drivers of case complexity, treatment planning appeared in priority competencies for both complexity due to child developmental history and complexity due to comorbid child psychopathology and dispositional factors. This focus on tailored treatment planning is consistent with literature highlighting the importance of formulation‐driven practice (Andrade et al., 2022; Day et al., 2011; Hawes & Dadds, 2021), and shows further support for modular models of intervention to which formulation and individual treatment planning is central (Chorpita et al., 2005; Weisz et al., 2012).

The current findings also emphasise the importance of process‐related competencies, beyond competencies related to specific PI skills, when case complexity stems from caregiver‐level and ecological‐factors. No priority competencies were related directly to the content of specific parenting skills or techniques in PIs for conduct problems. Rather, priority competencies for caregiver‐level and ecological drivers of case complexity most frequently concerned generic therapeutic competencies, that is, competencies common across all child and family therapy settings (Barker & Hawes, 2024). This focus on generic therapeutic competencies is consistent with longstanding evidence that common therapeutic factors, including therapeutic alliance, and agreed activities and goals, account for greater variance in outcomes than specific therapeutic techniques (Lambert & Barley, 2001). Literature on PIs specifically also supports this, emphasising the importance of therapeutic engagement competencies for programme effectiveness (Piotrowska et al., 2017). Indeed, a PI developed specifically for families with complex child conduct problems, the Helping Families Programme, focuses substantially on such competencies (Day et al., 2011). In light of previous research, the current findings suggest that the effective use of clinical process strategies is particularly critical for supporting children and families with complex needs.

The current model of drivers of case complexity and corresponding therapist competencies point to key priorities for future research to reduce the research‐practice gap highlighted in recent literature (Andrade et al., 2022). Most importantly, treatment process and outcome research is needed to better understand the processes by which these drivers of case complexity can affect treatment outcomes. We predict this may include at least four broad processes, the most common being disruption to parental engagement at any stage of treatment (e.g., attendance, implementation; Piotrowska et al., 2017). Secondly, when ecological drivers of case complexity also perpetuate the disruptive child behaviours (e.g., interparental conflict) they may continue doing so if not adequately addressed in an intervention; the same may be true for child‐level drivers associated with risk mechanisms not typically targeted in standard intervention (e.g., callous‐unemotional traits). Finally, therapists often respond to case complexity with treatment adaptations, which may improve outcomes for complex cases, but also have the potential to contribute to nonresponse when evidence‐based components are modified or removed by practitioners unnecessarily (Hawes & Dadds, 2021; Roach et al., 2022).

We predict that a consistent approach in research to characterising complex presentations, based on such a model, would enhance the quality of clinically applicable evidence available. Secondly, given that distinct competencies appear to be of key importance to these drivers, it is vital that intervention research addresses the significant heterogeneity that is known to characterise clinical populations of children with conduct problems (Andrade et al., 2022). Guidelines for adaptations based on this heterogeneity are nonetheless necessary to ensure their effectiveness (Hasson et al., 2023).

In clinical settings, consistent use of the current model to characterise complex presentations of conduct problems may support evidence‐based practice in various ways. Firstly, this model suggests a key focus be placed on the early identification and assessment, for each client, of the child and family factors represented by the respective drivers of case complexity. Likewise, these factors should be accounted for in formulation‐driven treatment planning, such that the intervention delivery is adapted to meet the unique needs of the family and optimise clinical engagement where possible. Furthermore, this model offers multidisciplinary teams a shared language to facilitate communication at the service‐level, where policy regarding referral, or the allocation of limited resources, may be based on criteria related to the complex needs of a family. Our findings likewise highlight that particular competencies may be beneficial to emphasise in practitioner training and supervision for complex client presentations, and that these may differ based on complexity profiles common to a particular service.

Our findings should be considered in light of some limitations. The current study focussed on the evidence‐based treatment of conduct problems in early‐to‐middle childhood and may not be representative of case complexity and associated competencies necessitated by presentations of conduct problems in older ages. Additionally, despite a broad international sample, participants largely represented Western, educated, industrialised, rich, democratic (WEIRD) countries. The research team aimed for a more heterogeneous sample, with contact attempts to practitioners in other countries either unsuccessful or limited by availability of contacts. Therefore, findings may not be generalisable to other population groups. Intervention research in more diverse populations remains a high priority for the field of child conduct problems (Fairchild et al., 2019; Hawes et al., 2023). Finally, while widely used to establish consensus in health research, the Delphi method may restate received wisdom that may or may not be supported by other empirical data. Therefore, further research should test the relative contribution of identified priority competencies to treatment outcomes for families with various complex needs. This could support the development of clinical practice guidelines to assist practitioners to make evidence‐based adaptations, which may differ according to different drivers of complexity.

There is much to be gained by adopting a consistent approach to characterising the complex needs of children with conduct problems in research and practice, and our findings have the potential to inform such an approach. An expert panel identified therapist competencies of key importance to seven distinct drivers of case complexity among children with conduct problems, thereby supporting the differentiation of these drivers in clinical training and decision‐making. The more that complexity arises from multiple drivers, especially those based in the family context and broader social environment of the child, the more likely it appears to be that optimal outcomes may rely on generic therapeutic competencies including process skills. There is a pressing need for coordinated, standardised, evidence‐based approaches to complex presentations of child conduct problems. The current findings can inform further research, including into the development and implementation of formal practice and training guidelines to better meet the complex needs of children with conduct problems in real‐world clinical settings.

## AUTHOR CONTRIBUTIONS

**Jessica M. Barker**: Conceptualization; data curation; formal analysis; investigation; methodology; project administration; visualization; writing—original draft; writing—review and editing. **David J. Hawes**: Conceptualization; investigation; methodology; supervision; writing—review and editing.

## CONFLICT OF INTEREST STATEMENT

The authors declare no conflicts of interest.

## ETHICS STATEMENT

Informed consent was appropriately obtained from all participants prior to their participation in this research study. Ethics approval was received on 16th March 2022 through the University of Sydney Human Research Ethics Committee (HREC; approval number 2022/HE000020).

## Data Availability

The data that support the findings of this study are available from the corresponding author upon reasonable request.
